# Formation of Advanced Glycation End Products during Frying of Potato Chips and Grilling of Beef Patties

**DOI:** 10.1021/acs.jafc.6c03413

**Published:** 2026-07-16

**Authors:** Thomas Heymann, Robert Rau, Marcus A. Glomb

**Affiliations:** Institute of Chemistry, Food Chemistry, 9176 Martin-Luther-University Halle-Wittenberg, Kurt-Mothes-Str. 2, 06120 Halle/Saale, Germany

**Keywords:** Maillard reaction, advanced glycation end products, frying, grilling, amide AGEs, pyridinium AGEs

## Abstract

Maillard reactions occur between reducing carbohydrates and mainly the *N* ^6^-amino group of lysine and the guanidino function of arginine, leading ultimately to advanced glycation end products (AGEs). In the present work, we give a comprehensive analysis of 15 AGEs in commercial potato chips, reaching a total of almost 5 mg/100 g chips, and study the change of protein modifications during the frying of potato slices. Major lysine protein modifications were *N* ^6^-carboxymethyl lysine, *N* ^6^-carboxyethyl lysine, pyrraline, and *N* ^6^-formyl lysine with contents of 500–950 μg/100 g chips. Additionally, we synthesized and detected *N* ^6^-(2,3-dihydroxypropyl)­lysine as a precursor probe for glyceraldehyde-derived AGEs and also quantitated 1-(5-amino-5-carboxy-pentyl)-3-(5-amino-5-carboxypentyl-amino) pyridinium salt (*meta*-DLP) and 2-ammonio-6-(3-oxidopyridinium-1-yl) hexanoate (OP-lysine) for the first time in a processed food matrix. These results were then transferred to the grilling of ribose-containing beef patties, where incubations of *N* ^2^-*t*-Boc-lysine with labeled 1-^13^C- and 5-^13^C-ribose gave mechanistic insights into the AGE-related degradation of the pentose.

## Graphical abstract

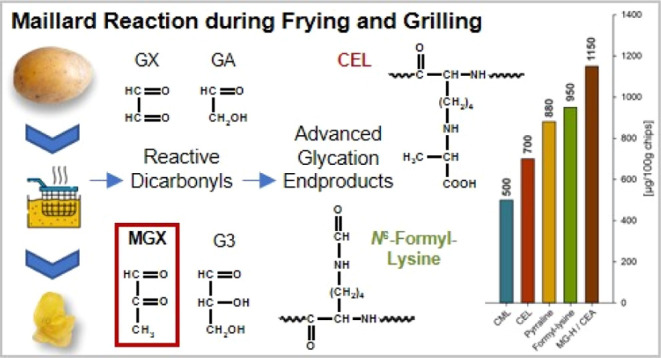

## Introduction

Thermal processing of foods such as baking, grilling, and frying initiates a number of chemical reactions. Among these, the so-called Maillard reaction is the most predominant and also the major source of aroma compounds, which are desirable in processed food. However, these nonenzymatic browning reactions also induce reactions of free amino acids or protein side chains with reducing carbohydrates, leading to a variety of stable and structurally heterogeneous protein adducts, named advanced glycation end products (AGEs).^1,2^ Early-stage reactions include Schiff base formation and subsequent rearrangement to Amadori products. These aminoketoses are prone to degrade in the advanced stages, giving rise to highly reactive carbonyl species. Especially 1,2-dicarbonyl compounds, such as glyoxal, react with nucleophilic protein side chains, leading to *N* ^6^-carboxymethyl lysine (**1**, CML, Figure 2), imidazolium cross-link glyoxal lysine dimer (**2**, GOLD), and amide structure *N* ^6^-glycoloyl lysine (**3**, GALA).^3^ The methylglyoxal lysine reaction cascade results in structurally analogous products, for instance, *N* ^6^-carboxyethyl lysine (**4**, CEL) and methylglyoxal lysine dimer (**5**, MOLD). Several studies showed that α-hydroxycarbonyls have a crucial impact on Maillard reactions, too, in particular on the formation of pyridinium AGEs.^4–6^ Recently, the spectrum of pyridinium AGEs was extended by a novel lysine–lysine cross-link.^7^ However, when it comes to specific information about the precursor imines detected as their corresponding reduction products, only *N* ^6^-(2-hydroxyethyl) lysine (**6**, HEL) for C_2_-imines has been well established in literature.^2,8^ Specifically, there is limited information about C_3_-imines.

During the heat treatment of foods, the levels of AGEs increase drastically. Typically, **1** (CML), **4** (CEL), and 5-hydroxymethylfurfural are often used as the markers of processed food. However, in view of the many established structures arising from Maillard reactions, the range must be significantly wider. From the nutritional aspects, the formation of AGEs may result in a reduced nutritional value of foodstuffs by losing essential amino acids. Also, the absorption of glycated proteins across the gastrointestinal mucosa is discussed in the literature after intestinal hydrolysis.^9^ The transepithelial transport of pyrraline (**7**, 6-(2-formyl-5-hydroxymethyl-1-pyrrolyl)-l-norleucine), a monovalent lysine modification originating from 3-deoxyglucosone, in peptide form via peptide transporters PEPT1 and PEPT2 into epithelial cells and ultimately into the bloodstream, is well understood.^10^ Thus, the quantitation of AGEs in processed food and the subsequent daily uptake of dietary AGEs are subject to current research.

In the present work, we give a comprehensive overview of Maillard-induced protein modifications in commercial potato chips and monitored their formation rates in a time-dependent manner during the frying of potato slices. Furthermore, we provide mechanistic insights into the degradation of ribose to the resulting AGEs during the grilling of beef patties with labeled ^13^C-ribose.

## Material and Methods

### Chemicals

All chemicals of the highest quality available were provided by Sigma-Aldrich (Munich/Steinheim, Germany), Iris-Biotech (Marktredwitz, Germany), Armar Chemicals (Leipzig, Doettingen, Germany), Roth (Karlsruhe, Germany), Merck (Darmstadt, Germany), ACROS Organics (Geel, Belgium), TCI Europe (Zwijndrecht, Belgium), Cambridge Isotope Laboratories (Tewksbury, USA), and VWR Chemicals (Darmstadt, Germany), unless otherwise indicated. The authentic reference materials *N* ^6^-carboxymethyl lysine (CML, **1**), *N* ^6^-(2-hydroxyethyl) lysine (HEL, **6**),^2^ *N* ^7^-carboxymethyl arginine (CMA, **15**),^11^ glyoxal hydroimidazolone (G-H3, **14**),^12^ N^6^-carboxyethyl lysine (CEL, **4**),^13^ glyoxal lysine dimer (GOLD, **2**),^3^ methylglyoxal lysine dimer (MOLD, **5**),^14^ *t*-butyl-2-[(*t*-butoxy-carbonyl) amino]-6-iodohexanoate (**C**),^15^ 4-(hydroxymethyl)­pyridin-3-ol (**A**), and 5-(hydroxymethyl)­pyridin-3-ol (**B**)^16^ were synthesized according to the literature.

### Synthesis of 2-Ammonio-6-[4-(hydroxymethyl)-3-oxidopyridinium-1-yl]­hexanoate (**9**, HOP-lysine)

The basic synthetic route was adapted from Argirov et al.^4^ Instead of starting with 4-picoline *N*-oxide, we used methyl 3-hydroxyisonicotinate and reduced the compound with lithium aluminum hydride to 4-(hydroxymethyl)­pyridin-3-ol **A** (Figure 1).

**Figure 1:**
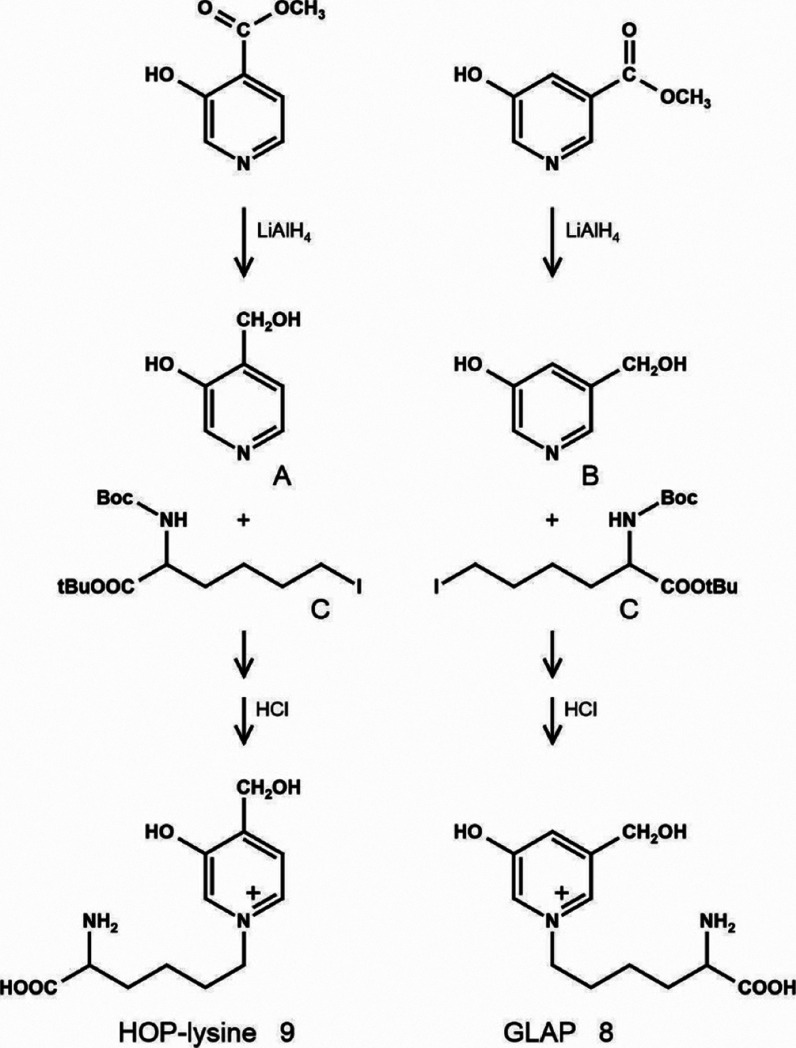
Synthesis of HOP-lysine **9** and GLAP **8**.

Compound **A** (104.6 mg, 0.5 mmol) was dissolved in 5 mL dry 1,4-dioxan under argon atmosphere and heated to 100 °C. Then, a solution of compound **C** (103.3 mg, 0.34 mmol) in 5 mL dry 1,4-dioxane was added dropwise. The mixture was stirred for 48 h at 100 °C. Solvents were removed under reduced pressure, and the crude product was treated with 10 mL of 3 M hydrochloric acid. The solution was stirred for 1 h at room temperature. Hydrochloric acid was evaporated under reduced pressure, and the residue was dissolved in 5 mL of water and extracted with diethyl ether (3 × 5 mL). The aqueous phase was evaporated to dryness, and the crude mixture was subjected to preparative HPLC (VYDAC 218TP1022, 250 × 20 mm, RP18, 10 μm, isocratic water/methanol 92:8 with 1.2 mL L^–1^ heptafluorobutyric acid (HFBA), 10 mL min^–1^). The apparatus consisted of a pump (Besta, Wilhelmsfeld, Germ any) with a degasser (Knauer, Berlin, Germany). Fractions containing HOP-lysine (*t*_R_, 31–39 min) were combined, solvents were evaporated, and the residue was lyophilized as a colorless amorphic material (10.4 mg, 0.041 mmol, 8%, HOP-lysine·4 HFBA). ^1^H NMR (400 MHz, D_2_O, δ (ppm)): 1.52 (m; 2H), 1.75 (m, 2H), 1.87 (m, 2H), 3.94 (t, ^3^ *J* = 6.0 Hz, 1H), 4.53 (t, ^3^ *J* = 7.0 Hz, 2H), 4.89 (s, 2H), 7.96 (d, ^3^ *J* = 6.2 Hz, 1H), 8.26 (d, ^3^ *J* = 6.2 Hz, 1H), 8.41 (s, 1H). ^13^C NMR (100 MHz, D_2_O, δ (ppm)): 23.6, 29.7, 31.3, 53.2, 59.5, 61.6, 124.6, 130.0, 137.3, 149.0, 154.9, 171.6. HR-MS, *m*/*z* 255.1340 (found) and *m*/*z* 255.1345 (calcd for C_12_H_19_O_4_N_2_ [M]^+^).

### Synthesis of 2-Ammonio-6-[5-(hydroxymethyl)-3-oxidopyridinium-1-yl]­hexanoate (**8**, GLAP)

The synthesis was performed as described above starting with 5-(hydroxymethyl)­pyridin-3-ol **B**. GLAP was purified by preparative RP-HPLC (VYDAC 218TP1022, 250 × 20 mm, RP18, 10 μm, isocratic water/methanol 95:5 with 1.2 mL L^–1^ HFBA, 10 mL min^–1^). Fractions containing GLAP (*t*_R_, 30–38 min) were combined, solvents evaporated, and the residue lyophilized as a colorless amorphic material (8.9 mg, 0.035 mmol, 7%, GLAP·4 HFBA). ^1^H NMR (400 MHz, D_2_O, δ (ppm)): 1.40 (m, 2H), 1.58 (m, 2H), 1.95 (m, 2H), 3.91 (t, ^3^ *J* = 6.1 Hz, 1H), 4.19 (t, ^3^ *J* = 6.9 Hz, 2H), 4.78 (s, 2H), 8.08 (s, 1H), 8.55 (s, 1H), 8.70 (s, 1H). ^13^C NMR (100 MHz, D_2_O, δ (ppm)): 21.5, 29.8, 30.1, 54.5, 60.7, 63.1, 125.5, 134.2, 138.1, 141.4, 155.9, 173.1. HR-MS, *m*/*z* 255.1340 (found) and *m*/*z* 255.1345 (calcd for C_12_H_19_O_4_N_2_ [M]^+^).

### Synthesis of *N* ^6^-(2,3-Dihydroxypropyl)­lysine (**19**, DPL)

273.9 mg (1.1 mmol) *N* ^2^-*t*-Boc-lysine, 143.2 mg (1.1 mmol) (*R*)-isopropylidenglyceraldehyde and 74.0 mg (1.2 mmol) sodium cyanoborohydride were dissolved in 7 mL of anhydrous methanol and stirred overnight at room temperature. Then, solvents were evaporated, and the resulting residue was subjected to column chromatography (silica gel, acetone/ethyl acetate/acetic acid, 1:2:0.1, v/v). Fractions containing material with *R*_f_ 0.30 (TLC, same eluent) were combined, eluents were removed under reduced pressure, and the residue was taken up in 3 M HCl and stirred for 30 min at room temperature. The solution was concentrated in vacuo and dried under a high vacuum to afford 65 mg (0.295 mmol, 27%) of DPL as a colorless powder. ^1^H NMR (400 MHz, CD_3_OD, δ (ppm)): 1.59 (m, 2H), 1.81 (m, 2H), 2.01 (m, 2H), 3.03 (dd, ^2^ *J* = 12.7 Hz, ^3^ *J* = 8.9 Hz, 1H), 3.09 (t, 2H), 3.20 (dd, ^2^ *J* = 12.7 Hz, ^3^ *J* = 4.1 Hz, 1H), 3.59 (m, 2H), 3.96 (m, 1H), 4.02 (t, ^3^ *J* = 7.2, 1H). ^13^C NMR (100 MHz, CD_3_OD, δ (ppm)): 23.2, 26.4, 30.9, 48.6, 51.5, 53.8, 65.1, 68.5, 171.6. HR-MS, *m*/*z* 221.2708 (found) and *m*/*z* 221.2714 (calcd for C_9_H_21_O_4_N_2_ [M + H]^+^).

### Model Incubations

Incubations were conducted in 0.1 M phosphate buffer at pH 7.4 and 37 °C in a shaker incubator (New Brunswick Scientific, Nürtingen, Germany) for 7 days under deaerated conditions. Deaerated conditions were achieved by the addition of 1 mM diethylenetriaminepentaacetic acid (DTPA). Buffer was degassed with helium before incubation. The samples were placed in 1 mL screw-cap vials without air and incubated under an argon atmosphere. *N* ^2^-*t*-Boc-lysine (42 mM) was incubated in the presence of glyoxal (42 mM), methylglyoxal (42 mM), glycolaldehyde (42 mM), glyceraldehyde (42 mM), or a mixture of glycolaldehyde (21 mM) and glyceraldehyde (21 mM). Sugar incubations contained 100 mM d-glucose, d-ribose, l-arabinose, d-xylose, d-threose, d-erythrose, and l-erythrulose. Model incubations with 1-^13^C- and 5-^13^C-labeled d-ribose (100 mM) were conducted under the same conditions. To stop incubations and for removal of the protection groups, the reaction mixtures were taken up in 3 M hydrochloric acid and kept for 30 min at room temperature. The acidic solvent was removed in a vacuum concentrator (Savant-Speed-Vac Plus SC 110 A combined with a Vapor Trap RVT 400, Thermo Fischer Scientific, Bremen, Germany), and the residue was redissolved and subjected to HPLC–MS/MS.

### Analytical HPLC-MS/MS

The HPLC apparatus (Jasco, Groβ-Umstadt, Germany) consisted of a pump (PU-2080 Plus) with a degasser (LG-2080-02) and a quaternary gradient mixer (LG-2080-04), a column oven (Jasco Jetstream II), and an autosampler (AS-2057 Plus). Mass spectrometric detection was conducted on an API 4000 QTrap LC–MS/MS system (AB Sciex, Concord, ON, Canada) equipped with a turbo ion spray source using electrospray ionization (ESI) in the positive mode (sprayer capillary voltage of 2.5 kV, nebulizing gas flow of 70 mL min^–1^, heating gas of 80 mL min^–1^ at 650 °C, and curtain gas of 40 mL min^–1^). The HPLC system was connected directly to the probe of the mass spectrometer. Nitrogen was used as the sheath and auxiliary gas. Chromatographic separations were performed on a stainless-steel column packed with the RP-18 material (Xselect HSS T3, 250 mm × 3.0 mm, 5 μm, Waters, Massachusetts) using a flow rate of 0.7 mL min^–1^. The mobile phase used consisted of ultrapure water (solvent A) and MeOH/water [7:3 (v/v), solvent B]. To both solvents (A and B), 1.2 mL L^–1^ of HFBA was added. For AGE analyses, the following gradient was used: 2% B isocratic for 2 min, to 14% B (in 10 min), to 87% B (in 22 min), to 100% B (in 0.5 min), and hold (7 min). Optimized mass spectrometric parameters and retention times (*t*_R_) for **9** (HOP-lysine), **8** (GLAP), and **19** (DPL) are summarized in Supporting Information Table SI1. Optimized parameters for MS for **3** (GALA), **5** (MOLD), **2** (GOLD), **15** (CMA), **13** (CEA), **6** (HEL), **7** (pyrraline), **1** (CML), **16** (*N* ^6^-formyl lysine), **17** (*N* ^6^-acetyl lysine), **11** (OP-lysine), **10** (*meta*-DLP), **14** (GH-3), and **12** (MGH-1/3 imidazolinones) were according to previous works.^7,17–19^ Quantitation was performed using the standard addition method. More precisely, increasing concentrations of authentic reference standards at factors 1, 1.5, and 2 times of the concentration of the analyte in the sample were added to aliquots of the sample.

### Commercial Potato Chips and Workup

Salted nonseasoned samples (*n* = 20) were purchased from local supermarkets. All samples were ground in a universal shredder (Moulinette, Tefal, Rumilly, France). Protein extraction was done as previously described by our working group.^7^ Complete defatting of samples was reached by a three times extraction and decantation with petroleum ether. Denovo formation of target compounds was checked and ruled out. Briefly, 2 g of defatted chips were dissolved in 15 mL of water. After adding 150 μL α-amylase (heat-stable, from *Bacillus licheniformis*), the reaction was heated to reflux for 30 min. Afterward, the solution was cooled down to 60 °C and 500 μL of amyloglucosidase (from *Aspergillus niger*, aqueous solution, >300 U/mL) was added prior to incubating for an additional 30 min. The solution was then cooled down to room temperature, and proteins were precipitated by adding 20 mL of −20 °C ethanol overnight in a freezer. The precipitate was centrifuged (9000*g*, 4 °C, 10 min), carefully washed twice with 15 mL of ice-cold water, and subsequently lyophilized. A total amount of 3 mg of the extracted protein was weighed in a 2 mL tube and homogenized in 1.5 mL of phosphate-buffered saline (PBS, 20 mM phosphate, 150 mM sodium chloride) using a mixer mill (Retsch MM400, Germany) combined with zirconium oxide grinding balls (5 mm) at 30 Hz for 45 min.

#### Acid Hydrolysis

400 μL of the above protein suspension was evaporated to dryness by vacuum centrifugation. The residue was treated with 200 μL of sodium borohydride (8 mg/mL) in sodium hydroxide (0.01 M) and incubated at room temperature for 1 h. The solution was evaporated to dryness again, and 800 μL of degassed 6 M HCl was added. The mixture was heated for 20 h at 110 °C under an argon atmosphere in a drying oven. Afterward, solvents were evaporated by vacuum centrifugation, and the residue was dissolved in 492 μL of 0.05 M HCl. For furosine analysis, the reduction step was omitted. In selected cases for differentiation of HEL-1D and HEL-2D, sodium borodeuterate was used for reduction.

#### Enzymatic Hydrolysis

Protein isolate extracted from commercial chip samples was pooled and enzymatically hydrolyzed. In 2 mL tubes, 1.5 mL of PBS was added to 3 mg of the pooled samples and homogenized in a mixer mill as described above. To 400 μL of the protein suspension, the following proteases were added every 24 h: 0.5 units of papain, again 0.5 units of papaine, 0.3 units of Pronase E, again 0.3 units of Pronase E, 1.0 unit of leucine aminopeptidase, and 0.95 units of carboxypeptidase Y. A small crystal of thymol was added with the first addition of enzyme. Samples were incubated at 37 °C in a shaker incubator for 96 h. After completion of the last digestion step, the reaction mixture was filtered through a 3000 Da molecular weight cutoff filter (VWR International, Radnor, PA).

### Determination of Extraction Yield and Protein Purity

The protein content of the commercial chips and the residue were analyzed by the Kjeldahl method. 1.0 g of minced sample was weighed into a Kjeldahl flask and digested with concentrated sulfuric acid at 370 °C for 90 min in the presence of a selenium catalyst (Wieninger catalyst, Merck, Germany; Tecator Digester 2520, Foss, Denmark). The solution was cooled down to room temperature, and 20 mL of water and 20 mL of sodium hydroxide solution (32%) were added to the flask. By distillation, ammonia was transferred into a sulfuric acid solution (0.05 M). After adding seven drops of Tashiro’s indicator, the solution was titrated with sodium hydroxide solution (0.10 M). For calculation of total protein, the conversion factor 6.25 was used.

### Preparation and Frying of Potato Slices

A batch (1 kg) of potatoes (cultivar Karelia) was purchased at a local supermarket. Potato tubes were immediately washed, peeled, and cut by using a vegetable slicer into 1 mm thick slices. Slices were treated with distilled water to remove starch from the surface. Then, 200 g of potato slices were put into a vessel with a heating basket, which was large enough to allow free movement of the slices in the frying oil. The potato slices were fried at 160 °C for 2, 5, 8, 11, and 16 min in 2.5 L of vegetable oil (sunflower oil, Brökelmann + Co, Hamm, Germany). The frying process was repeated three times with fresh potato slices and fresh oil. Then, fried potato slices were dried over a wire screen and cooled to room temperature. Afterward, the chips were homogenized using a Moulinette and subsequently lyophilized. Fat extraction was done by repetitive addition of petroleum ether, followed by ultrasonic extraction for an additional 15 min and centrifugation (8000*g*, 10 min).

### Meat Samples and Workup

Preparation of meat samples was done as previously described by Eggen and Glomb^20^ Fresh minced beef samples (3 × 400 g) were purchased at a regional supermarket and pooled. Then, patties (Ø 8 cm, thickness 1.5 cm) were formed with 130 g of meat and grilled for 6 min using an electric indoor grill (Tefal Optigrill GC705D, Group SEB, Frankfurt, Germany). Each time point was done with three separate samples. Patties were grilled on both sides at 230 °C. The surface temperatures of the griddles were measured by using an infrared thermometer (TFA Dostmann, Wertheim, Germany). The meat samples were cooled down to room temperature, homogenized using a Moulinette and lyophilized. Defatting was achieved by the addition of petroleum ether, following ultrasonic extraction (15 min) and centrifugation (8000*g*, 10 min). This extraction step was repeated five times; afterward, the samples were dried and stored at −18 °C.

### Analysis of Ribose

In a 15 mL centrifuge tube, 500 mg of a defatted and lyophilized raw meat sample was weighed and homogenized in 5.5 mL of water/methanol (81:19, v/v) using an Ultra-Turrax (Ika, Germany). After adding 1 mL of 50% (w/w) trichloroacetic acid, the protein content was precipitated and centrifugated (8000*g*, 10 min, 4 °C). The supernatant was filtered through a syringe filter (0.45 μm, cellulose acetate, Carl Roth AG, Germany), and the filtrate was derivatized according to Rakete and Glomb.^21^ In general, 150 μL of the filtrate was mixed with 100 μL of 1-naphthylamine solution (0.2 M in dimethyl sulfoxide (DMSO)/15% acetic acid 1:1, v/v) and 500 μL of sodium cyanoborohydride solution (1.0 M in DMSO), incubated for 16 h at room temperature, and then subjected to HPLC-FLD.

### HPLC-FLD

Analyses were carried out on a Jasco HPLC system consisting of a pump (PU-980) with a degasser (DG-2080-53), a quaternary gradient mixer (LG-2080-02), a column oven (Jetstream II), an autosampler (851-AS), and an FLD (FP-4020). Chromatographic separations were performed on a stainless steel column (Knauer, Eurospher 100-5 C18, 250 × 4.6 mm, RP18, 5 μm, Hesperia, CA) using a flow rate of 1.0 mL L^–1^ and a column temperature of 25 °C. The excitation was adjusted to 318 nm and the emission to 440 nm. Eluents were water (A) and a mixture of methanol and ultrapure water (70:30, v/v; B). HFBA (0.6 mL L^–1^) was added to both eluents as an ion pair reagent. Samples were injected at 35% B (75 min isocratic) to 100% B (in 5 min) and hold (15 min).

### High-Resolution Mass Determination (HR-MS)

Positive ion high-resolution ESI mass spectra were obtained from an Orbitrap Elite mass spectrometer (Thermofisher Scientific, Germany) equipped with a heated ESI electrospray ion source (spray voltage 3.5 kV; capillary temperature 275 °C, source heater temperature 40 °C; FTMS resolution >30,000). Nitrogen was used as a sheath and auxiliary gas. The sample solutions were introduced continuously via a 500 μL Hamilton syringe pump with a flow rate of 5 μL/min. The data were evaluated by the Xcalibur software 2.7 SP1.

### Nuclear Magnetic Resonance Spectroscopy (NMR)

NMR spectra were recorded on a VXR 400 spectrometer (Varian, Palo Alto) operating at 400 MHz for ^1^H and 100 MHz for ^13^C. Tetramethylsilane was used as a reference for calibrating the chemical shifts.

### Statistical Analysis

All significance tests were performed by two-sample *t*-test with a probability value of 95%, 99%, and 99.9%. The WELCH test was used alternatively in case of differing standard deviations. The same probability value was selected. Statistical evaluation was performed by the use of the software SigmaPlot (version 14.0 build 14.0.3.192, Systat Software Inc.).

## Results and Discussion

### Formation of Pyridinium AGEs in Deaerated and Aerated Model Incubations with Reducing Sugars

Incubations of short-chain carbonyls with *N* ^2^-*t*-Boc-lysine were performed in phosphate buffer pH 7.4 at 37 °C for 7 days. LC–MS/MS chromatograms of aerated and deaerated incubations demonstrated the exclusive formation of 2-ammonio-6-[3-(hydroxy-methyl)-3-oxidopyridinium-1-yl]­hexanoate (**8**, GLAP) by glyceraldehyde (G3) and the formation of 2-ammonio-6-[4-(hydroxymethyl)-3-oxidopyridinium-1-yl]­hexanoate (**9**, HOP-lysine) by glycolaldehyde (GA) (Table 1, Supporting Information Table SI2, Figure 2).

**Table 1:** Formation of GLAP (**8**), HOP-Lysine (**9**), and CML (**1**) in *N* ^2^-*t*-Boc-Lysine (42 mM) Incubations with Carbonyl Compounds (42 mM, 7 d, 37 °C, pH 7.4, Aeration/Deaeration): Glycolaldehyde (GA), Glyceraldehyde (G3), Glyoxal (GX), and Methylglyoxal (MGX)

incubation	CML	GLAP	HOP-lysine
	[mmol/mol lysine]	[μmol/mol lysine]	
GA (aerated)	38.4 ± 1.8	<LOD	116 ± 10
GA (deaerated)	4.9 ± 0.3	<LOD	214 ± 15
GA/G3 (aerated)	16.2 ± 0.8	7.9 ± 0.6	16.4 ± 1.2
GA/G3 (deaerated)	2.5 ± 0.1	8.3 ± 0.7	28.6 ± 2.2
G3 (aerated)	<LOD	39.0 ± 2.1	<LOD
G3 (deaerated)	<LOD	40.2 ± 3.8	<LOD
GX (aerated)	43.3 ± 1.4	<LOD	<LOD
GX (deaerated)	42.9 ± 1.2	<LOD	<LOD
MGX (aerated)	<LOD	<LOD	<LOD
MGX (deaerated)	<LOD	<LOD	<LOD

**Figure 2:**
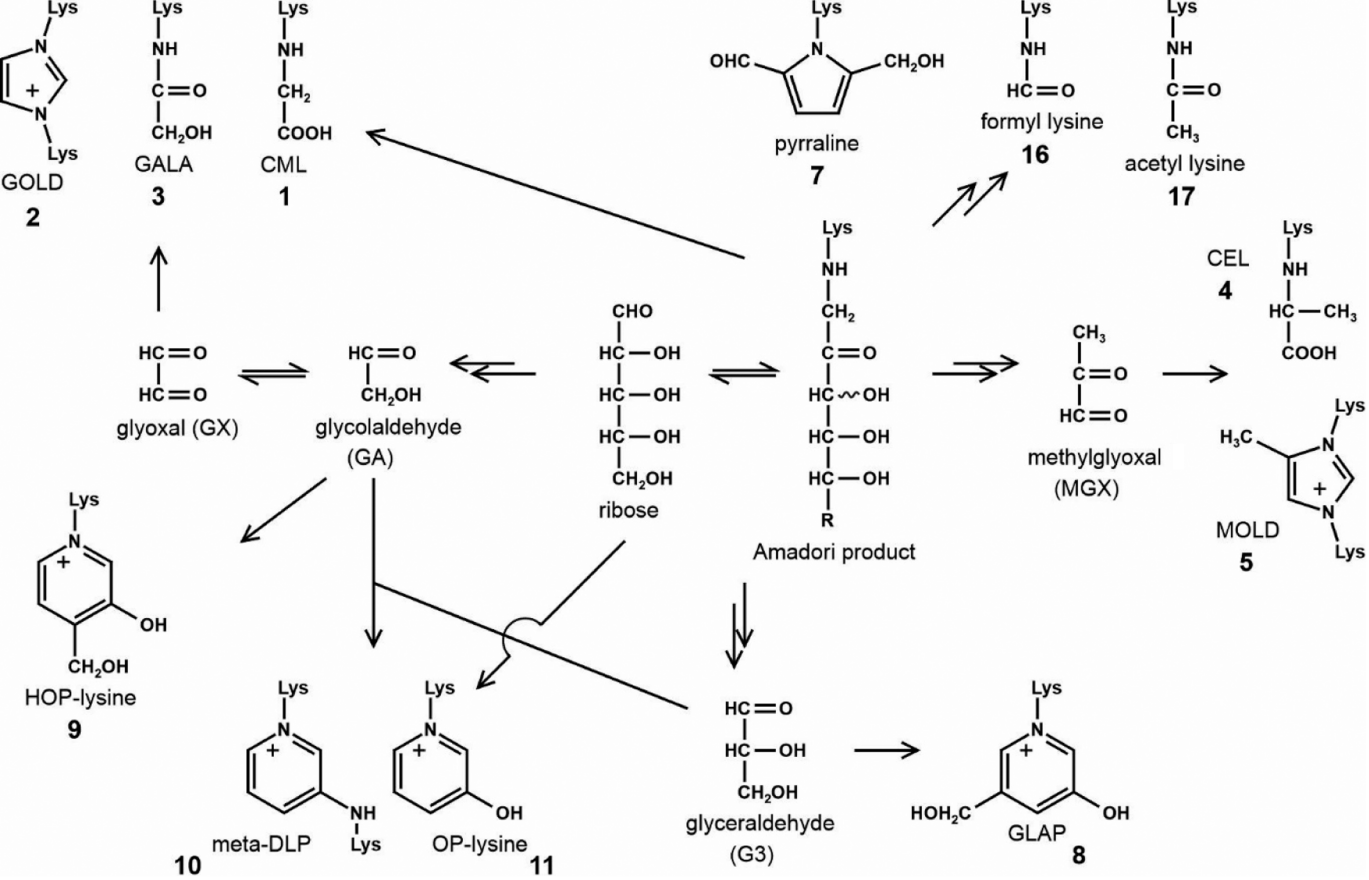
Structures and mechanistic correlations of lysine AGEs discussed; ribose R = H and glucose R = CH_2_OH.

Interestingly, although **9** (HOP-lysine) and **8** (GLAP) as constitutional isomers have very similar chemical structures, completely different precursor structures lead to the formation of these pyridinium AGEs (Figure 2). After a 7 day incubation with GA under nonoxidative conditions, **9** (HOP-lysine) reached 214 μmol/mol lysine and nearly 2-fold lower amounts in aerated GA incubation, respectively. This is due to the oxidation of GA to glyoxal (GX) under oxidative conditions in the presence of lysine, which was confirmed by the significantly enhanced formation of *N* ^6^-carboxymethyl lysine (**1**, CML). In contrast, the formation of **8** (GLAP) showed no significant difference between aerated and deaerated conditions in all incubations. Combined GA/G3 incubations resulted in lower amounts for **9** (HOP-lysine) and **8** (GLAP). In addition, incubations of *N* ^2^-*t*-Boc-lysine with glyoxal (GX) or methylglyoxal (MGX) showed no formation of both pyridinium AGEs, underlining that GA and G3 are the only short-chain precursor structures for **9** (HOP-lysine) and **8** (GLAP), respectively.

To gain evidence for **9** (HOP-lysine) and **8** (GLAP) formation during Maillard-induced fragmentation of sugars, we incubated different carbohydrates with *N* ^2^-*t*-Boc-lysine under the same conditions as described above. Since GA is an important precursor structure for **9** (HOP-lysine) and to prevent the oxidation of GA to GX, incubations with higher-chained sugars were conducted under nonoxidative conditions. Indeed, on the basis of the data in Table SI3 (Supporting Information), aerated incubations led to significantly lower amounts of **9** (HOP-lysine). As summarized in Table 2 8 (GLAP) was only detected in ribose and in aerated ascorbic acid incubations. In contrast, **9** (HOP-lysine) was formed in all C_4_- and C_5_-sugar incubations. In general, the C_4_-carbohydrates d-threose, d-erythrose, and l-erythrulose led to higher yields of **9** (HOP-lysine), reaching up to 140, 41, and 139 μmol/mol lysine, respectively. This is in line with the findings by Argirov et al., however, who provided only qualitative evidence of **9** (HOP-lysine). A possible formation pathway for **9** (HOP-lysine) was studied in depth by that workgroup.^4^ Interestingly, our results now suggest that this pathway is driven by stereochemical aspects. The mechanistic insights from model incubations were then transferred to potato chips and grilled beef patties, where reactive carbonyl compounds stem from starch degradation, lipid peroxidation, and ribose-releasing metabolites, respectively.

**Table 2:** Formation of GLAP (**8**), HOP-Lysine (**9**) and CML (**1**) in *N* ^2^-*t*-Boc-Lysine (42 mM) Incubations with Reducing Sugars (100 mM, 7 d, 37 °C, pH 7.4, Aeration/Deaeration)

incubation	CML	GLAP	HOP-lysine
	[mmol/mol lysine]	[μmol/mol lysine]	
d-glucose (deaerated)	0.19 ± 0.14	<LOQ	<LOQ
d-ribose (deaerated)	13.8 ± 0.9	0.14 ± 0.01	14.3 ± 1.1
d-threose (deaerated)	6.8 ± 0.4	<LOQ	140 ± 9
d-erythrose (deaerated)	6.7 ± 0.4	<LOQ	41 ± 2
l-erythrulose (deaerated)	7.2 ± 0.5	<LOQ	139 ± 9
ascorbic acid (deaerated)	<LOD	<LOD	<LOD
ascorbic acid (aerated)	7.9 ± 0.5	0.14 ± 0.01	3.6 ± 0.3

### Extraction of Potato Chips Protein and Determination of AGE Levels in Commercial Chips

Frying is one of the oldest heat processing methods. Nevertheless, fried potato products such as French fries and chips are consumed worldwide and increasingly represent an important part of the modern Western diet. With the use of fat as a heating medium, temperatures reaching up to 180 °C are standard. This favors Maillard reactions and the formation of protein modifications. In the present work, we give an overview of 15 AGEs and 4 other amino acid modifications, with three pyridinium AGEs being quantified for the first time in chips using high-performance liquid chromatography-tandem mass spectrometry (HPLC–MS/MS). In total, 20 unseasoned commercial chips were obtained from local super markets and analyzed in triplicate. Unseasoned chips were chosen to exclude any impact of ingredients other than potatoes on AGE formation and also to ensure compatibility with model frying experiments. First, potato proteins were isolated from the other chip ingredients according to an established protocol for high-starch foods containing steps of defatting and enzymatic starch hydrolysis. Potato chips are mainly composed of starch (∼50%), followed by fat (∼35%) and proteins (∼6%). The remainder is salt (∼1%) and dietary fiber (∼2%). Complete extraction of potato proteins is challenging due to the insoluble proteins that build the cell walls.^22^ The protein isolation resulted in yields between 60% and 70%, and the purity (50%)—representing the protein content of the obtained precipitates—was calculated based on the nitrogen content, which was determined by the Kjeldahl method. These results are comparable with the findings by Waglay and Karboune investigating the isolation of proteins from potato pulp.^23^ Next, potato proteins were reduced with sodium borohydride and acid-hydrolyzed. However, to get a broad overview of Maillard-induced protein modifications, it is indispensable to quantitate acid-labile structures. Therefore, the 20 isolated proteins were pooled and hydrolyzed enzymatically without reduction. The efficiency of the enzymatic digestion was calculated based on the acid-stable *N* ^6^-carboxyethyl lysine (**4**, CEL) and was about 40%. This moderate yield was within our expectations, as hydrolysis rates decrease with an increase of thermal treatment, as reported, e.g., for the baking of wheat dough.^19^ Thus, by definition, the content of **4** (CEL) from acid hydrolysis was set to 100% and was used to adjust the quantitation of acid labile AGEs. **1** (CML) was used as a second marker for the enzymatic hydrolysis correction factor to reevaluate the calculations based on **4** (CEL). Due to the incomplete protein extraction and possible partial determination of protein modifications, the original chip samples were defatted and directly acid-hydrolyzed. The detected value of **1** (CML) showed virtually no difference in comparison with the amounts quantitated in the protein isolates. Therefore, it has to be stated that although the protein extraction was limited to 60–70%, the exact content of protein modification could be safely quantitated.

The overall results of acid-stable protein modifications are shown in Figure 3 (Supporting Information Table SI4).

**Figure 3:**
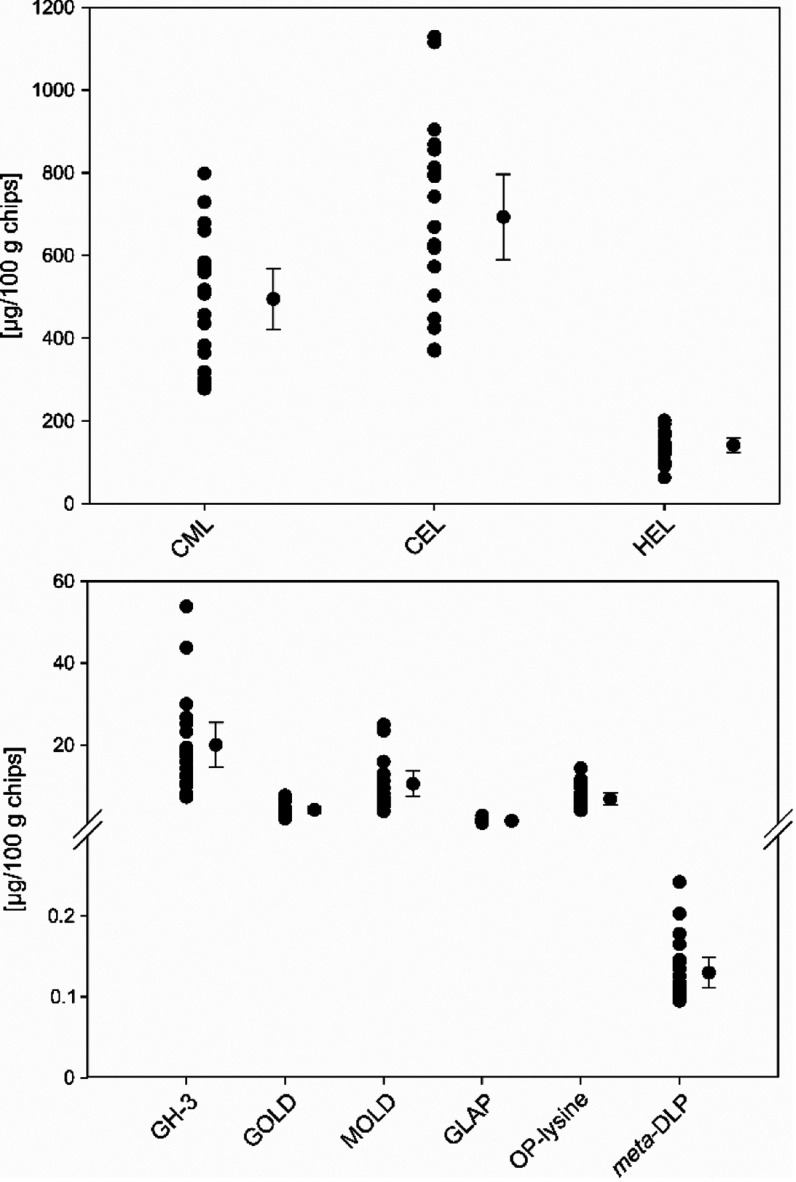
Acid-stable AGE levels in commercial potato chips. HOP-lysine was below LOQ.

MGX-derived **4** (CEL) was a dominant AGE structure with an amount of 700 μg/100 g chips, GX-related **1** (CML) gave 500 μg/100 g chips. These results were comparable with the broad ranges in previous reports between 24 and 2400 μg/100 g for **4** (CEL) and 15 to 1500 μg/100 g for **1** (CML).^24–27^ The ratio of **4** (CEL) to **1** (CML) was 1.4:1, which is in line with the nearly 3-fold higher levels of **5** (MOLD) compared to the GX-analogous **2** (GOLD) (10.5 vs 4.2 μg/100 g). However, it has to be mentioned that **1** (CML) also results from oxidative fragmentation reactions of Amadori products (Figure 2). Hence, this suggests that MGX is the leading precursor for AGE formation in fried potatoes. This is also reflected in the literature, detecting 2-fold higher amounts of MGX compared to GX,^28,29^ although some authors declare nearly the opposite.^30^ Ledbetter et al. showed that the soaking pretreatment of potatoes before frying had a decreasing effect on the formation of dicarbonyls, especially for MGX.^25^ Recently, a novel pyridinium structure, namely, *meta*-DLP (**10**) was identified in hydrolyzed chip protein.^7^ Here, we quantitated this compound for the first time in the food matrix with amounts between 0.10 and 0.24 μg/100 g chips (mean of 20 samples: 0.13 ± 0.04 μg/100 g). Although the structural class of pyridinium-AGEs has been studied in model incubations and in vivo, specific information about formation in processed foods is lacking. Besides pentosidine, only G3-derived **8** (GLAP) was identified in heated food.^31,32^ In the present work, **8** (GLAP) and **11** (OP-lysine) were detected in commercial chips, giving values of about 1.5 μg/100 g and 6.9 μg/100 g, respectively. However, GA-derived **9** (HOP-lysine) was below LOQ.

The MGX-hydroimidazolones MG-H (**12**, MG-H3, and MG-H1 are given as a sum parameter) and their open-chained form *N* ^7^-carboxyethyl arginine (**13**, CEA) were the predominant arginine modifications, found at 500 and 650 μg/100 g, respectively (Table 3, Figure SI1 Supporting Information). Arginine-bound GX was estimated by the quantitation of G-H3 (**14**) in acidic workup at about 20 μg/100 g. **14** (G-H3) is formed by 5-(4,5-dihydroxy-2-imino-1-imidazolidinyl)­norvaline (dihydroxyimidazolidine) and *N* ^7^-carboxymethyl arginine (**15**, CMA) under the harsh conditions of acid protein hydrolysis and can therefore be regarded as a sum parameter for both structures.^12^ Again, MGX-derived AGEs were found at higher levels than the GX-analogues. Pyrraline (**7**) was the second most abundant protein modification, reaching levels of about 880 μg/100 g. This lysine-derived pyrrol-AGE has been reported several times for heated foods and is the major protein modification in baked bread rolls.^19,33,34^ Because **7** (pyrraline) is fully reconverted to lysine during acid hydrolysis, this analyte can only be accessed by enzymatic hydrolysis.^35^ Interestingly, the most important AGE in fried potatoes was *N* ^6^-formyl lysine (**16**) with values of about 950 μg/100 g. Moreover, *N* ^6^-acetyl lysine (**17**) was also of quantitative relevance, found at 500 μg/100 g. **16** (*N* ^6^-formyl lysine) has been mechanistically related to glucosone and maltosone via β-fragmentation, while **17** (*N* ^6^-acetyl lysine) originates likewise from 1-deoxyglucosone, respectively.^19^ However, in the case of **17** (*N* ^6^-acetyl lysine), this is paralleled by enzymatic biochemical acetylation and deacetylation pathways.^36^

**Table 3:** Concentration of Acid-Labile AGEs and *o*-Tyrosine as an Oxidative Marker in Pooled Potato Chips Protein Released by Enzymatic Hydrolysis

		[μg/100 g chips]
AGE	CEA **13**	650 ± 60
	MG-H1/H3 **12**	500 ± 50
	pyrraline **7**	880 ± 80
	GALA **3**	130 ± 20
	*N* ^6^-formyl lysine **16**	950 ± 90
	*N* ^6^-acetyl lysine **17**	500 ± 50
oxidative marker	*o*-tyrosine	182 ± 20

### Determination of AGEs during the Frying Process of Potato Chips

To gain further insights into the formation of AGEs during deep frying, the process was monitored in a time-resolved manner. Potato slices were fried for 2, 5, 8, 11, and 16 min, then allowed to cool to room temperature, subsequently defatted and acid hydrolyzed, and analyzed with HPLC–MS/MS. Figure 4 shows the time course formation of furosine (**18**), **4** (CEL), **1** (CML), and pyridinium AGEs.

**Figure 4:**
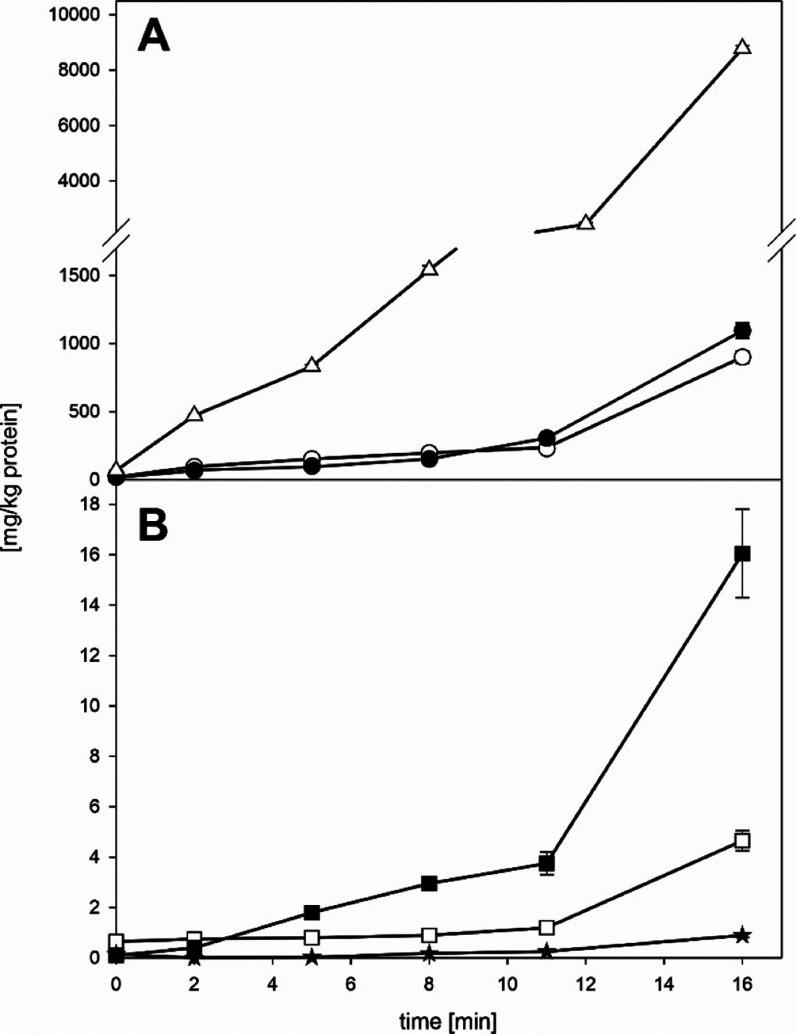
Time-dependent formation of AGEs in potato chips during deep-frying (160 °C). (A) △ furosine **18**, ● CEL (**4**), and ○ CML (**1**). (B) ■ OP-lysine (**11**), □ GLAP (**8**), and ★ *meta*-DLP (**10**).

After 11 min, a pronounced increase of all analytes can be observed. It has to be noted that after this time period, the browning of the crust also evoked. Former investigations showed that browning strongly correlates to water loss and that the Maillard reaction in foods primarily depends on the moisture content.^19,37^ Indeed, the present frying time appeared to be particularly long, since in the literature the moisture content decreased from 80% to <2% in less than 6 min at 140 °C.^38^ However, this also strongly depends on the thickness of the potato slices, the temperature, and the material-to-oil ratio. For the present experiment, color formation at 11 min was best comparable to commercial chips.

In general, the levels of AGEs increased continuously and rose strongly during the last minutes. The amounts of **1** (CML) detected in raw potato slices were 19 mg/kg of protein, and frying led to an increase to 901 mg/kg of protein. Similar formation rates were also determined for **4** (CEL). The final detected amounts for both AGEs and the **1** (CML)-to-**4** (CEL) ratio (1:1.2) were in line with the results of the commercial chips described above. The evaluation of the early stages of the Maillard reaction can be assessed by quantitation of **18** (furosine), which is formed during acid hydrolysis of Amadori compounds by 40 mol % (Figure SI2, Supporting Information).^39^ **18** (furosine) was the most abundant modification found in fried potatoes. Interestingly, amounts increased starting from 65 mg/kg protein in raw potato slices to about 8750 mg/kg protein at 16 min. Unexpectedly, no degradation was observed at any time. This might be due to the progressing degradation of starch to glucose, maltose, and oligosaccharides, which continuously provides reducing sugars for Amadori product formation. Thus, ongoing oxidative Amadori degradation might be an additional source of **1** (CML) formation. When it comes to quantitation of pyridinium AGEs, **8** (GLAP), **11** (OP-lysine), and **10** (*meta*-DLP) were determined. Levels of **8** (GLAP) and **10** (*meta*-DLP) quasi remained unchanged during the first minutes, while **11** (OP-lysine) rose from 0.10 mg/kg protein to 3.75 mg/kg protein at 11 min. However, in the last 4 min of frying, the amount of all pyridinium AGEs increased nearly fourfold.

To get deeper mechanistic insights into the relationship between precursor structures and protein modifications, protein-bound GA/GX as *N* ^6^-(2-hydroxyethyl) lysine (**6**, HEL) and protein-bound G3 as *N* ^6^-(2,3-dihydroxypropyl) lysine (**19**, DPL), respectively, were monitored after reduction (Figure 5, Figure SI2 Supporting Information).

**Figure 5:**
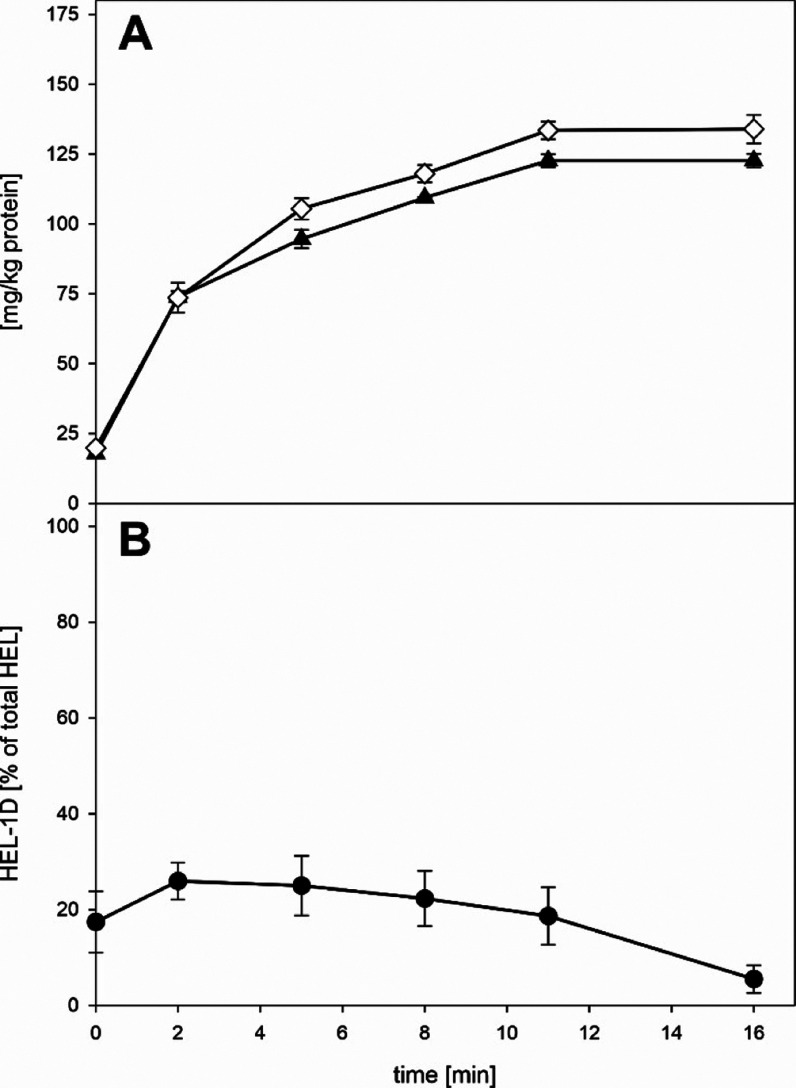
Time-dependent formation of DPL (**19**, ◇), HEL (**6**, ▲) (A), and *N* ^6^-(2-hydroxy-1-*d*-ethyl)-lysine (HEL-1D) relative to total content of HEL (B) in potato chips during frying (160 °C).

**19** (DPL) was synthesized as an authentic reference standard analogous to **6** (HEL) via reductive amination.^2^ As yet, little attention has been paid to G3-derived protein modification.^40^ On the other hand, in literature α-dicarbonyl compounds such as GX or MGX are often determined in full recovery as their respective quinoxalines after derivatization with *o*-phenylenediamine.^41^ However, for GA and G3 the resulting benzimidazols yielded only recoveries of 38% and 76%, respectively.^42^ A recent approach by Martin-Morales et al. using 1-phenyl-3methyl-5-pyrazolone led to higher recoveries for G3.^43^ Here, we detected the formed imines as their corresponding reduction products, which also allowed us to differentiate between GX- and GA-imines.^8^ Specifically, the reduction with sodium borodeuterate leads to *N* ^6^-(2-hydroxy-1,2-*d*-ethyl)­lysine (HEL-2D) for GX-imine and *N* ^6^-(2-hydroxy-1-*d*-ethyl)­lysine (HEL-1D) for GA-imine.

Results for **6** (HEL) and **19** (DPL) are displayed in Figure 5A. Both structures had similar formation rates and rose from 20 mg/kg protein to about 130 mg/kg protein within 11 min, and then, a plateau was reached. The detection of **19** (DPL) proves the formation of reactive G3 during the frying process. However, the resulting imines represent no stable end product. In fact, model incubations with *N* ^2^-*t*-Boc-lysine under moderate conditions showed imine degradation, monitored as **6** (HEL) already after 3 h.^8^ Thus, in the present investigation, degradation and formation of **6** (HEL) and **19** (DPL) in the last minutes of frying must be equal. The formed imines degrade with reactive carbonyl compounds to stable end products, which is in line with the increase in AGEs after 11 min as described above. HEL-1D, illustrated as the percentage of total **6** (HEL) (sum of HEL-1D and HEL-2D), reached 23% after 2 min and decreased over the time period continuously to 7% (Figure 5B). The oxidation of GA to GX obviously proceeds very efficiently during frying. Interestingly, although GA-imine was present in detectable amounts, levels of **9** (HOP-lysine) were too low for quantitation, in line with above commercial potato chip analyses. As demonstrated in previous works, the formation of GA and GX as a result of lipid peroxidation is minor compared to formation by Maillard reactions once carbohydrates and proteins are present.^44^

### Determination of Advanced Glycation End Products in Meat

On the basis of the above model incubations ribose was identified as a potent precursor structure for the formation of pyridinium AGEs; thus, with a focus on acid-stable structures, the change of protein modifications in beef meat was investigated during grilling.^7^ According to literature, the main carbohydrates in beef meat are glucose, glucose-6-phosphate and, to smaller extent, ribose.^45^ Here, the detected amounts of ribose in raw ground beef were 0.41 mmol/kg meat. The course of AGE formation during grilling (230 °C) of beef patties is displayed in Table 4 (Supporting Information SI-5). **4** (CEL) was the predominant protein modification with 9.1 mg/kg protein for raw meat, which increased by a factor of about 4 to 31.7 mg/kg at 6 min. Similar formation rates were observed for **1** (CML) with amounts of 7.3 mg/kg protein in raw meat, and grilling led to an increase to 27.0 mg/kg. Surprisingly, **11** (OP-lysine) was present already in raw meat with 22.8 μg/kg protein and only gave a nonsignificant trend to 24.2 μg/kg when heated. As expected from the aerated model experiments **9** (HOP-lysine) was not quantifiable. To demonstrate that ribose is indeed an important precursor structure for AGE formation, it was added to the patties 10-fold the amounts occurring naturally. This led, after grilling, to double the formation of **1** (CML) and **4** (CEL) and almost tripled the amounts of **11** (OP-lysine). Now, also **10** (*meta*-DLP) and **9** (HOP-lysine) showed up at 3.2 μg/kg and 14.2 μg/kg protein, respectively, while **8** (GLAP) stayed below LOQ. This was in line with a major 3-fold increase of **6** (HEL) for C_2_-precursors GA/GX. Although not relevant for beef, l-erythrulose was added in a different experiment for mechanistic reasons. As expected from the above lysine carbohydrate model incubations (Table 2), this led to a major increase of **9** (HOP-lysine) to 60.3 μg/kg protein, while all other AGE parameters stayed almost the same as native heat treatment.

**Table 4:** Formation of AGEs and HEL/DPL in Native and d-Ribose or l-Erythrulose Added Beef Patties During Grilling (230 °C)^a^

	raw	native	d-ribose	l-erythrulose
	0 min	6 min	6 min	6 min
[mg/kg protein]				
CML **1**	7.3 ± 0.7	27.0 ± 1.7***	55.9 ± 2.6***	33.2 ± 1.8**
CEL **4**	9.1 ± 0.9	31.7 ± 3.0***	68.1 ± 3.3***	34.0 ± 2.1
HEL **6**	5.9 ± 0.5	17.0 ± 1.1***	57.9 ± 1.0***	19.8 ± 1.4
DPL **19**	<LOD	<LOQ	<LOQ	<LOQ
[μg/kg protein]				
GLAP **8**	<LOD	<LOQ	<LOQ	<LOD
OP-lysine **11**	22.8 ± 1.5	24.2 ± 1.2	67.6 ± 3.6***	24.7 ± 1.7
*meta*-DLP **10**	<LOD	<LOD	3.3 ± 0.3	<LOD
HOP-lysine **9**	<LOD	<LOQ	14.2 ± 1.8	60.3 ± 3.5

a Level of significance: ** α = 1%, *** α = 0.1%, native vs raw, ribose or erythrulose vs native.

Next, to understand the impact of specific fragmentation precursors for AGE formation, 1-^13^C- and 5-^13^C-labeled ribose were incubated with *N* ^2^-*t*-Boc-lysine under moderate and deaerated conditions (Table 5). The two pathways of **1** (CML) formation, oxidative cleavage of the Amadori product and the GX-lysine isomerization cascade, were confirmed (Figure 2). By definition, in 1-^13^C-reactions, the Amadori origin must lead to the incorporation of the label, while the nonlabeled part must be attributed to the GX reaction. **6** (HEL) can be used to dissect the total GX/GA contribution and shows that about 10% incorporated the label and, thus, stem from the 1-C to 2-C-region, i.e., for **1** (CML) formation, this means that 67% (70–3% for the GX reaction at 1-C–2-C) were formed via the Amadori product cleavage and 31% (28 + 3%) via GX. This was fully supported by the 5-^13^C reaction with vice-versa ratios. As MGX is specific for the formation of **4** (CEL), the labeled ribose incubations gave a ratio of 4:6 for this three-carbon fragment from the 1-C-3-C to the 3-C–5-C part of the carbohydrate skeleton.

**Table 5:** Proportion of Isotopologues from the Reaction between *N* ^2^-*t*-Boc-Lysine, 1-^13^C-Ribose, or 5-^13^C-Ribose (100 mM, 7 d, 37 °C, pH 7.4, Deaeration)

analyte	*m*/*z* (M^+^)	1-^13^C-ribose [%]				5-^13^C-ribose [%]			
		0^a^	1	2	3	0	1	2	3
CML **1**	205.1	28.3	69.5	<1	<1	71.8	26.9	<1	<1
CEL **4**	219.1	58.5	40.6	<1	<1	43.1	57.1	<1	<1
HEL **6**	191.2	91.5	8.2	<1	<1	82.7	17.0	<1	<1
HOP-lysine **9**	255.3	68.3	27.9	3.7	<1	<1	35.6	59.1	<1
OP-lysine **11**	225.3	1.6	97.3	<1	<1	1.7	98.3	<1	<1
*meta*-DLP **10**	353.3	2.5	92.7	2.2	2.6	3.0	95.6	2.5	<1

a The number of ^13^C atoms in the molecule; the natural isotope effect was corrected.

Argirov et al. established two mechanisms leading to **9** (HOP-lysine) based on stable isotope labels and NMR experiments.^4^ First, solely GA reactions lead to **9** (HOP-lysine), which means that three GA molecules must participate to build up the C_6_-pyridinium moiety. For the present 1-^13^C-ribose incubation, this would entail a maximum of three labels being incorporated, however, which was not verified. In addition, in the 5-^13^C-reaction, no unlabeled **9** (HOP-lysine) was found, despite the information from **6** (HEL) that major parts of C_2_-fragments stem from the region 2-C to 4-C. Taken together this concludes that a solely GA-based formation is of minor, if any, importance. Instead, the label distribution confirmed the predominant reaction via the second mechanism, combining a C4-fragment and GA. While Argirov et al. argued based on ascorbic acid degradation, here erythrulose can be formed via hydrolytic β-fragmentation from ribose to release formic acid. This explains why in 1-^13^C-reactions major parts of **9** (HOP-lysine) are not labeled; as for erythrulose formation, 1-^13^C is cleaved off, and reaction with nonlabeled and labeled GA leads to the present isotopic contribution. This also explains that with 5-^13^C no unlabeled **9** (HOP-lysine) was found, as, by definition, the C_4_-part always includes the label.

For **11** (OP-lysine), the formation was originally proposed based on the reaction of GA and G3 with lysine based on a di-Amadori compound intermediate to give the C_5_-pyridinium aromatic structure. Indeed, the formation of G3 from xylose was reported via hydrolytic β-dicarbonyl cleavage from 1-deoxypentosone under release of acetic acid.^46^ Later, we were able to establish an alternative pathway solely from interacting two C_2_-carbonyl compounds, GA and GX, under scission of a C_1_-fragment.^7^ The participation of two or three fragments would mean for the ribose experiments herein that incorporation of two up to three labels should be found. Instead, an almost exclusive one-label implementation was found in 1-^13^C as well as in 5-^13^C reactions. As this cannot be explained via reactions of fragments, this strongly suggested that the intact carbon backbone as such reacts with lysine to give **11** (OP-lysine). This represents a third mechanism of formation and challenges the concept of a di-Amadori intermediate. To unequivocally establish the mechanism, labeled experiments with preparative isolation of target structures should be conducted to site-specifically locate the incorporation of the label by NMR in follow-up investigations.

In summary, the present report provides a comprehensive analysis of the AGE content of potato chips. Methylglyoxal-related lysine and arginine AGEs (CEL **4**, CEA **13**, MG-H **12**) totaled up to 1850 μg/100 g chips, followed by **7** (pyrraline) and **1** (CML) (880 and 500 μg/100 g). Unexpectedly, the amide AGE **16** (*N* ^6^-formyl lysine) was the single major modification with 950 μg/100 g, the importance of which should be deepened in future investigations. For the first time the pyridinium AGEs **11** (OP-lysine), **10** (*meta*-DLP), and **9** (HOP-lysine) were quantitated in foods, expanding the AGE profile in potato chips and grilled beef patties. The importance of glyceraldehyde for the formation of pyridinium AGEs **11** (OP-lysine), **8** (GLAP), and **10** (*meta*-DLP) was confirmed by identification and correlation of the respective lysine-imine in potato chips. For **11** (OP-lysine), a novel mechanistic route from ribose was established incorporating the whole carbon backbone.

## Supplementary Material



## Notes

Structures and mechanistic correlations of arginine AGEs discussed; ribose R = H and glucose R = CH_2_OH; probes to analyze Maillard intermediates: HEL (**6**) after reduction for the imine of lysine with glyoxal/glycolaldehyde; DPL (**19**) after reduction for the imine of lysine with glyceraldehde; furosine (**18**) after acid hydrolysis for the Amadori product; optimized mass spectrometric parameters and retention times for HOP-lysine (**9**), GLAP (**8**), and DPL (**19**); LOD/LOQ for AGEs and DPL/HEL analysis in *N* ^2^-*t*-Boc-lysine incubations; formation of GLAP (**8**), HOP-lysine (**9**), and CML (**1**) in *N* ^2^-*t*-Boc-lysine (42 mM) incubations with reducing sugars (100 mM, 7d, 37 °C, pH 7.4, aeration); LOD/LOQ for AGEs and other amino acid modifications analysis in potato chips; and LOD/LOQ for AGEs and HEL/DPL analysis in beef patties (PDF)

## Abbrevation Used

AGE: advanced glycation end products
CEA: *N* ^7^-carboxyethyl arginine
CEL: *N* ^6^-carboxyethyl lysine
CMA: *N* ^7^-carboxymethyl arginine
CML: *N* ^6^ carboxymethyl lysine
DPL: *N* ^6^-(2,3-dihydroxypropyl)­lysine
DTPA: diethylenetriaminepentaacetic acid
GA: glycolaldehyde
GALA: *N* ^6^-glycolyl lysine
G-H3: glyoxal hydroimidazolone
GLAP: 2-ammonio-6-[3-(hydroxymethyl)-3-oxidopyridinium-1-yl] hexanoate
GOLD: glyoxal lysine derived dimer
GX: glyoxal
G3: glyceraldehyde
HEL: *N* ^6^-(2-hydroxyethyl)­lysine
HEL 1D: *N* ^6^-(2-hydroxy-1-*d*-ethyl)­lysine
HEL 2D: *N* ^6^-(2-hydroxy-1,2-*d*-ethyl)­lysine
HOP-lysine: 2-ammonio-6-[4-(hydroxymethyl)-3-oxidopyridinium-1-yl]­hexanoate
*meta*-DLP: 1-(5-amino-5-carboxypentyl)-3-(5-amino-5-carboxy-pentylamino) pyridinium salt
MGX: methylglyoxal
MOLD: methylglyoxal lysine dimer
OP-lysine: 2-ammonio-6-(3-oxidopyridinium-1-yl) hexanoate
