# Supplementary material for: Formation of Advanced Glycation End Products during Frying of Potato Chips and Grilling of Beef Patties
Source: J Agric Food Chem. 2026 Jul 16;74(29):23081–91. doi: 10.1021/acs.jafc.6c03413 (PMC13426301; doi:10.1021/acs.jafc.6c03413)
Supplement: Supplementary file 1 [file jf6c03413_si_001.pdf]

# Supporting Information

Formation of Advanced Glycation End Products during Frying of Potato Chips and Grilling of Beef Patties

Thomas Heymann, Robert Rau and Marcus A. Glomb*

Institute of Chemistry, Food Chemistry, Martin-Luther-University Halle-Wittenberg, Kurt-Mothes-Str. 2, 06120 Halle/Saale, Germany

*To whom correspondence should be addressed (e-mail marcus.glomb@chemie.uni-halle.de, Fax ++049-345-5527341)

FIGURES

**Figure SI-1.** Structures and mechanistic correlations of arginine AGEs discussed; ribose R=H, glucose R=$CH_2OH$.

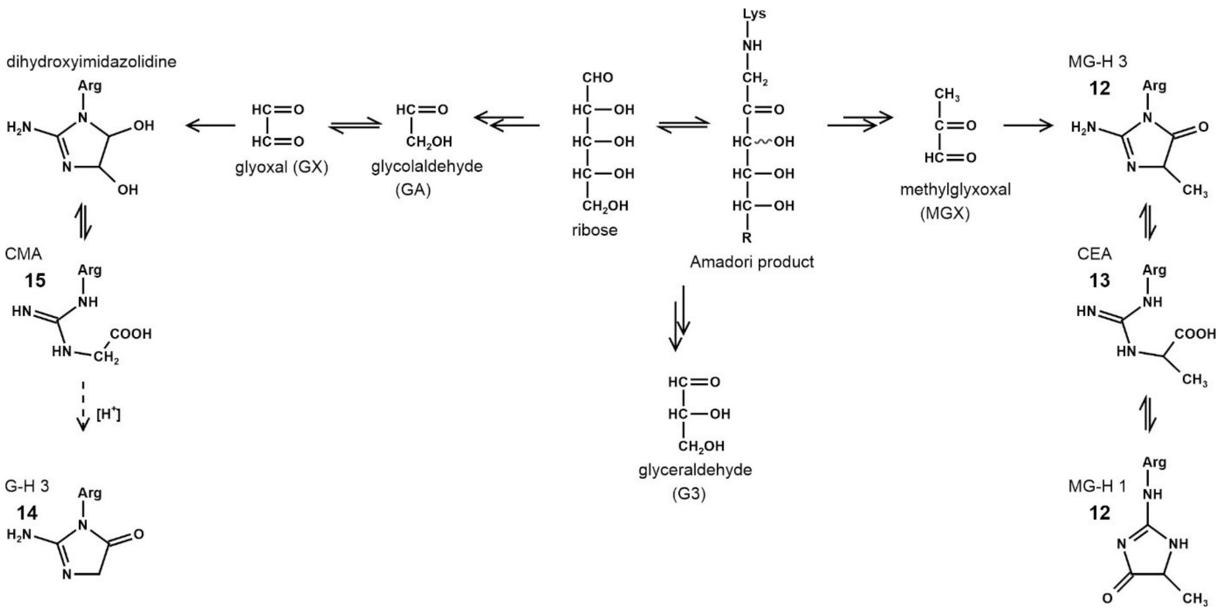


**Figure SI-2.** Probes to analyze Maillard intermediates: HEL (**6**) after reduction for the imine of lysine with glyoxal/glycolaldehyde; DPL (**19**) after reduction for the imine of lysine with glyceraldehde; furosine (**18**) after acid hydrolysis for the Amadori product.

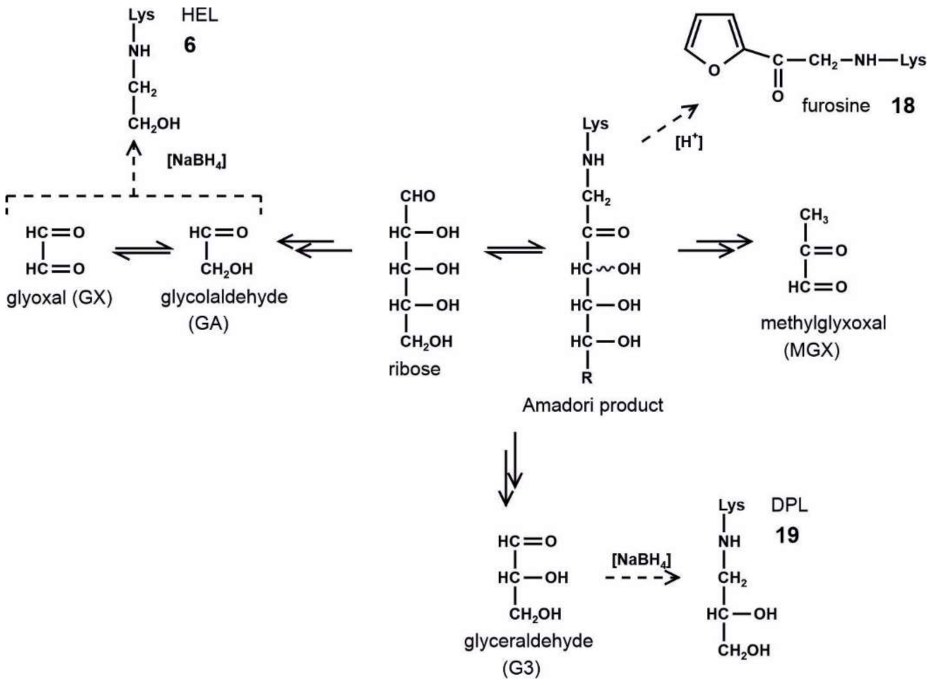

# TABLES

**Table SI-1.** Optimized Mass Spectrometric Parameters and Retention Times for HOP-Lysine (**9**), GLAP (**8**) and DPL (**19**).

| | Fragments [m/z] | declustering potential [V] | collision energy [eV] | cell exit potential [V] |
|---|---|---|---|---|
| HOP-lysine | QN 255.3/84.3 | 70 | 40.0 | 16.0 |
| ($t_R$ = 23.3 min) | QL 255.3/130.3 | 70 | 22.0 | 15.0 |
| | QL 255.3/210.3 | 70 | 25.0 | 12.0 |
| GLAP | QN 255.3/84.3 | 70 | 35.0 | 13.0 |
| ($t_R$ = 22.7 min) | QL 255.3/130.3 | 70 | 23.0 | 10.0 |
| | QL 255.3/210.3 | 70 | 27.0 | 14.0 |
| DPL | QN 221.3/84.3 | 45 | 37.0 | 14.0 |
| ($t_R$ = 13.5 min) | QL 221.3/130.3 | 45 | 22.0 | 11.0 |
| | QL 221.3/210.3 | 45 | 30.0 | 12.0 |

QN = quantifier, QL = qualifier

**Table SI-2.** LOD/LOQ for AGEs and DPL/HEL Analysis in $N^2$-*t*-Boc-Lysine Incubations.

| | LOD [µmol/mol lysine] | LOQ |
|---|---|---|
| CML **1** | 0.7 | 2.1 |
| CEL **4** | 0.6 | 1.9 |
| DPL **19** | 0.7 | 2.1 |
| GLAP **8** | 0.1 | 0.4 |
| HEL **6** | 0.7 | 2.1 |
| HOP-lysine **9** | 0.1 | 0.4 |
| *meta*-DLP **10** | 0.1 | 0.4 |
| OP-lysine **11** | 0.2 | 0.6 |

**Table SI-3.** Formation of GLAP (**8**), HOP-Lysine (**9**) and CML (**1**) in $N^2$-*t*-Boc-Lysine (42 mM) Incubations with Reducing Sugars (100 mM, 7d, 37 °C, pH 7.4, Aeration).

| Incubation | CML | GLAP | HOP-lysine |
|---|---|---|---|
| | [mmol/mol lysine] | [µmol/mol lysine] | |
| D-glucose (aerated) | 1.4 ± 0.1 | < *LOQ* | < *LOQ* |
| D-ribose (aerated) | 55.5 ± 1.8 | 0.12 ± 0.01 | 2.4 ± 0.2 |
| D-threose (aerated) | 11.1 ± 0.6 | < *LOQ* | 73 ± 3 |
| D-erythrose (aerated) | 12.0 ± 0.7 | < *LOQ* | 20 ± 1 |
| L-erythrulose (aerated) | 9.9 ± 0.5 | < *LOQ* | 75 ± 9 |

**Table SI-4.** LOD/LOQ for AGEs and other Amino Acid Modifications Analysis in Potato Chips.

| | LOD | LOQ |
|---|---|---|
| | [mg/kg protein] | |
| AGEs | | |
| CEA **13** | 0.06 | 0.18 |
| MG-H3/H1 **12** | 0.07 | 0.24 |
| G-H3 **14** | 0.12 | 0.38 |
| CML **1** | 0.05 | 0.16 |
| CEL **4** | 0.04 | 0.13 |
| GOLD **2** | 0.04 | 0.12 |
| GLAP **8** | 0.01 | 0.03 |
| GALA **3** | 0.03 | 0.09 |
| HOP-lysine **9** | 0.01 | 0.03 |
| *meta*-DLP **10** | 0.01 | 0.03 |
| MOLD **5** | 0.03 | 0.10 |
| OP-lysine **11** | 0.01 | 0.04 |
| pyrraline **7** | 0.05 | 0.15 |
| $N^6$-formyl lysine **16** | 0.07 | 0.20 |
| $N^6$-acetyl lysine **17** | 0.06 | 0.18 |
| other modifications | | |
| furosine **18** | 0.04 | 0.12 |
| HEL **6** | 0.04 | 0.12 |
| DPL **19** | 0.06 | 0.18 |
| *o*-tyrosine | 0.06 | 0.19 |

**Table SI-5.** LOD/LOQ for AGEs and HEL/DPL Analysis in Beef Patties.

| | LOD | LOQ |
|---|---|---|
| [mg/kg protein] | | |
| CML **1** | 0.6 | 1.9 |
| CEL **4** | 1.2 | 3.7 |
| HEL **6** | 0.9 | 2.8 |
| DPL **19** | 3.7 | 11.1 |
| [µg/kg protein] | | |
| GLAP **8** | 2.2 | 6.5 |
| OP-lysine **11** | 1.4 | 4.3 |
| *meta*-DLP **10** | 1.0 | 3.0 |
| HOP-lysine **9** | 3.0 | 8.9 |
